# Germline Variants in Cancer Predisposition Genes in Pediatric Patients with Central Nervous System Tumors

**DOI:** 10.3390/ijms242417387

**Published:** 2023-12-12

**Authors:** Aleksa Jovanović, Nataša Tošić, Irena Marjanović, Jovana Komazec, Branka Zukić, Marina Nikitović, Rosanda Ilić, Danica Grujičić, Dragana Janić, Sonja Pavlović

**Affiliations:** 1 Pediatric Oncology Department, National Cancer Research Center, 11000 Belgrade, Serbia; aleksa.jovanovic@ncrc.ac.rs (A.J.); dragana.janic@ncrc.ac.rs (D.J.); 2 Laboratory for Molecular Biomedicine, Institute of Molecular Genetics and Genetic Engineering, University of Belgrade, 11042 Belgrade, Serbia; natasa.tosic@imgge.bg.ac.rs (N.T.); irena.marjanovic@imgge.bg.ac.rs (I.M.); jovana.komazec@imgge.bg.ac.rs (J.K.); branka.zukic@imgge.bg.ac.rs (B.Z.); 3 Pediatric Radiation Oncology Department, National Cancer Research Center, 11000 Belgrade, Serbia; marina.nikitovic@ncrc.ac.rs; 4 Faculty of Medicine, University of Belgrade, 11000 Belgrade, Serbia; rosandailic@gmail.com (R.I.); gruj59@gmail.com (D.G.); 5 Neurooncology Department, Neurosurgery Clinic, University Clinical Centre of Serbia, 11000 Belgrade, Serbia

**Keywords:** childhood cancer, central nervous system tumors, genomic alterations, targeted therapy

## Abstract

Central nervous system (CNS) tumors comprise around 20% of childhood malignancies. Germline variants in cancer predisposition genes (CPGs) are found in approximately 10% of pediatric patients with CNS tumors. This study aimed to characterize variants in CPGs in pediatric patients with CNS tumors and correlate these findings with clinically relevant data. Genomic DNA was isolated from the peripheral blood of 51 pediatric patients and further analyzed by the next-generation sequencing approach. Bioinformatic analysis was done using an “in-house” gene list panel, which included 144 genes related to pediatric brain tumors, and the gene list panel Neoplasm (HP:0002664). Our study found that 27% of pediatric patients with CNS tumors have a germline variant in some of the known CPGs, like *ALK*, *APC*, *CHEK2*, *ELP1*, *MLH1*, *MSH2*, *NF1*, *NF2* and *TP53*. This study represents the first comprehensive evaluation of germline variants in pediatric patients with CNS tumors in the Western Balkans region. Our results indicate the necessity of genomic research to reveal the genetic basis of pediatric CNS tumors, as well as to define targets for the application and development of innovative therapeutics that form the basis of the upcoming era of personalized medicine.

## 1. Introduction

Central nervous system (CNS) tumors comprise around 20% of all childhood malignancies. Despite many improvements, the survival of affected patients is still unsatisfactory, and residual long-term neurological sequelae represent a significant concern [1,2]. In order to more successfully diagnose and treat these diseases, a better understanding of their pathogenetics has become a necessity.

Wide use of next-generation sequencing (NGS) in oncology significantly contributed to unveiling the genetics behind tumor pathology and directed the development of targeted therapies. Aside from defining novel variants in cancer tissue, NGS contributed to a better understanding of germline variants harboring risk for cancer occurrence [3,4].

Germline variants in cancer predisposition genes (CPGs) are found in approximately 10% of pediatric neuro-oncology patients. In particular tumor types such as atypical teratoid rhabdoid tumors, choroid plexus carcinomas and medulloblastomas, these germline variants are registered even more frequently [5]. However, the genetic background of predisposition for the development of pediatric CNS tumors has been insufficiently characterized compared to other types of childhood cancers.

From the genetic point of view, tumorigenesis is a multistage process, and according to Knudson’s two-hit theory, the germline variants in CPGs represent the initial, “first-hit” variation, and tumor development occurs only after the “second-hit” change, which can be limited only to the tumor tissue [6]. In this regard, the detection of germline variants can contribute to the detection of individual pediatric patients with a predisposition, not only to primary CNS tumors but also to the development of secondary CNS tumors that occur after the treatment of other types of tumors. Namely, the burden of secondary malignancies after cancer treatment in childhood is not negligible [7], and information on existing germline variants in CPGs in these patients can guide clinical decision-making to minimize this risk and provide optimal treatment [8,9,10,11].

In order to identify patients who should be referred to a clinical geneticist and genetic counseling, pediatricians today are relying on several guidelines, none of which are specific solely to CNS tumors [12,13]. This study aimed to determine the spectrum of germline variants in 144 CPGs in 51 pediatric patients with CNS tumors and to investigate whether these variants are associated with distinct clinical characteristics.

## 2. Results

This study encompassed 51 pediatric patients diagnosed with CNS tumors in a two-year period. The median age was 8 (4, 14) years with slight male predominance (57%). Patient characteristics are represented in Table 1.

High-grade gliomas were the most prevalent tumor type, comprising almost one-third (29%) of all cases. Medulloblastomas (21%) and low-grade gliomas (16%) were also very frequent. There were five patients (10%) who did not undergo biopsy; therefore, the pathology of their tumor remained unknown. The MRI imaging implied that three patients had diffuse intrinsic pontine gliomas, one had optic pathway glioma and one patient had a suspected secondary tumor in the brainstem, but in this case, medulloblastoma dissemination could not be ruled out without a biopsy. Tumor types with lower prevalence were ependymomas (6%), atypical teratoid rhabdoid tumors (4%) and choroid plexus tumors (4%). Additionally, we detected one patient with craniopharyngioma, one with germ cell tumor, one with diffuse glioneuronal leptomeningeal tumor and one patient with composite ependymoma/low-grade glioma tumor. Also, one patient had multiple syndromic tumors (ependymomas, vestibular schwannomas, meningiomas).

Three out of 51 patients (6%) have been diagnosed and treated for secondary brain tumors. Two of them had confirmed high-grade glioma, and one did not have confirmed tumor pathology. All three of them were previously treated with both radiotherapy and chemotherapy. Lethal outcome was observed in seven patients, and three of them (#4, #5 and #6) carried germline variants in CPGs.

Although the entire cohort of 51 patients underwent genetic screening for germline variants, in parallel we evaluated widely used guidelines for assessing the need for genetic testing—the Jongmans selection tool and McGill Interactive Pediatric OncoGenetic Guidelines (MIPOGG). The Jongmans tool recognized 12 out of 14 patients with germline variants, missing 1 patient with high-grade glioma and 1 with ependymoma. Similarly, the MIPPOG tool also recognized 12/14 patients with germline mutations, not targeting 1 patient with medulloblastoma and the other with ependymoma, which has been missed by the Jongmans tool as well.

The total diagnostic interval was 8 (3, 16) weeks with no statistically significant difference between the patients with germline variants (11.5 (2.75, 32.75) weeks) and patients without them (8 (3, 14) weeks).

**Figure 1:**
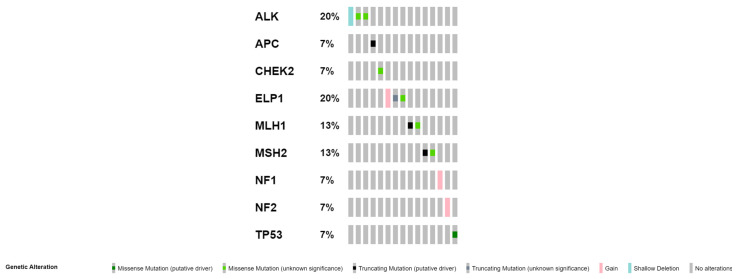
OncoPrint showing the distribution of germline genetic alterations in cancer predisposition genes in 14 patients. The types of mutations are labeled in the color legend, particular genes in rows, and tumor samples in columns. The ninth and tenth columns correspond to one patient (tumor) labeled as 9 [14].

Variants found in examined patients and relevant clinical data for all of them are reported in Table 2. In 14 out of 51 patients (27%), we identified a total of 15 germline variants. Among the detected 15 variants, 4 were (27%) pathogenic (P), 6 (40%) likely pathogenic (LP) and 5 (33%) variants of uncertain significance (VUS). Seven variants were not reported previously, among which 4 were likely pathogenic and 3 were VUS. Three novel variants were detected in the *ELP1* gene, 3 in the *ALK* gene and 1 in the *MSH2* gene. All detected variants were heterozygous and there were 2 frameshift indels, 6 nonsense variants, and 7 missense variants (Figure 1). Germline variants in the *ELP1* gene have been associated with pilocytic astrocytoma for the first time.

Twelve out of 14 patients with detected germline variants had a positive family history. Patient #1 with medulloblastoma and *ALK* germline variant had a cousin with a brain tumor in childhood. Patient #2 with the same diagnosis and *ALK* variant had a great-grandfather with lung cancer in advanced age and a great-uncle with laryngeal cancer in the forties. The father of patient #4 (high-grade glioma already treated for medulloblastoma, and *APC* variant) was diagnosed with familial adenomatous polyposis, highly indicative of Turcot’s syndrome. Patient #5 with suspected Li–Fraumeni syndrome was diagnosed with medulloblastoma at an early age and a *CHEK2* variant. From the mother’s side, this patient had a grandfather with lymphoma, a great-grandfather with unspecified abdominal cancer and from the father’s side a grandfather with prostatic cancer. Patients #6 and #7 with *ELP1* variants both had grandparents with leukemia and colorectal cancer, respectively. In patient #8 diagnosed with pilocytic astrocytoma and *ELP1* germline variant, six relatives with unknown tumors in the father’s family were reported. The father of patient #9 with high-grade glioma and two heterozygous *MLH1* variants died of colorectal carcinoma, while the grandfather of patient #11 with high-grade glioma and *MSH2* variant had a positive history on the mother’s side of the family, with a grandfather who died from stomach cancer and a grandmother from a brain tumor. Patient #12 with neurofibromatosis type 1 had multiple ancestors from the mother’s side of the family with fibromas and optic pathway gliomas.

Pancreatic cancer was noted in the grandmother of patient #13 with neurofibromatosis type 2. The mother of patient #14 with *TP53* germline mutation had a benign colon polyp at a young age, while her mother had breast cancer in her twenties, which is indicative of potential Li–Fraumeni syndrome.

Two of the patients with germline variants in *APC* (patient #4) and *ELP1* (patient #6) had secondary malignancy. One patient with *ALK* variant (#2) and medulloblastoma suffered from postoperative mutism (posterior fossa syndrome). Patient #7 with *ELP1* variant had excessive toxicity to chemotherapy (severe vincristine neuropathy). Based on clinical information (tumor types, café au lait spots), patients #12 and #13 were diagnosed with neurofibromatosis type 1 (NF1) and type 2 (NF2), respectively. Skin changes were observed in mentioned patients with *ELP1* (#7), *NF1* (#12) and *NF2* (#13) variants as well as in patient #9 with composite heterozygous *MLH1* variants.

According to the current standard treatment protocols, almost all the children received radiation therapy. It was only avoided in a patient with NF1 (#12). All but one patient (#10) were treated with systemic therapy. Two of them received targeted therapy (patient #8 with low-grade glioma and proven pathogenic variant in *BRAF* gene in the tumor tissue received MEK and BRAF inhibitors; patient #13 with NF2 was treated by VEGF and mTOR inhibition due to the known genetic disease) contrasted to other patients who received conventional chemotherapy.

## 3. Discussion

In this study, we determined the frequency and spectrum of germline variants in 144 cancer-associated genes in 51 pediatric patients with CNS tumors and investigated whether these variants are associated with distinct clinical characteristics.

It was previously observed that patients with neurocutaneous syndromes spent more time between the onset of symptoms and definitive diagnosis than patients without them in our country, even though a prompt approach is suggested by diagnostic protocols. Therefore, it was notable to evaluate the presence of other cancer-predisposing syndromes in this population and their total diagnostic interval (TDI). Even though there was no statistically significant difference considering TDI in patients with and without variants in tumor-predisposing genes (11.5 vs. 8 weeks), this period was longer in patients with the found variants. This supports the view that the wariness of tumor risk in patients with neurocutaneous and other predisposing syndromes is not high among practitioners in our country and requires further education to raise awareness of the topic [15]. To overcome this problem, tools like the one by Jongmans et al. and MIPOGG were developed. Their value for physicians was previously proven [13,16,17]. Our results support these findings, with both MIPOGG and the Jongmans tool selecting 12 out of 14 with mutated predisposing genes. Nevertheless, Jongmans tool proved to be more specific. Additionally, Jongmans criteria were updated to include any tumor suggestive of genetic syndrome, and particular high-grade glioma types were recently connected with CPGs. Considering these updates, all of our positive patients would have been selected with Jongmans tool [4,18].

Our study found that 27% of pediatric patients with CNS tumors have a variant in some of the germline-predisposing genes. It is generally considered that variants in CPGs are found in around 10% of patients [5]. Most recent studies of germline predisposition in pediatric CNS tumors report this number to range from 9% to 18% (Table 3) [19,20,21].

These differences are attributable to ethnic variations, sample size and biased sampling. The higher frequency of germline variants detected in our study might also be influenced by these factors. Even though our research covered all patients in our national tertiary center, children who are considered disease-free after surgery (around 40% of patients with primary CNS tumors—mostly craniopharyngiomas, low-grade gliomas and ependymomas) do not get referred to our institution. However, new CPGs are being recognized, and their real prevalence in pediatric neuro-oncology patients is probably greater than 10%.

We discovered variants in nine genes: *ALK*, *APC*, *CHEK2*, *ELP1*, *MLH1*, *MSH2*, *NF1*, *NF2* and *TP53*. Four out of seven novel variants that we detected are classified as LP and need to be functionally characterized to be proven to be clinically relevant. The other three variants are VUS; therefore, additional data and functional characterization are needed for clear evidence of their clinical impact.

### 3.1. ALK Gene

The anaplastic lymphoma kinase (ALK) is a tyrosine kinase receptor mainly expressed in neural tissues during embryonic development, but also in neuroblastoma, the most common extracranial childhood solid tumor [22]. Somatic and germline gene aberrations, leading to ALK activation, are also present in this disease [23] and were reported in familial and sporadic neuroblastoma [24]. Passoni et al. found that ALK overexpression is associated with advanced/metastatic neuroblastoma [25], and it has been suggested that high levels of mutated and wild-type ALK mediate similar molecular pathways that may contribute to a malignant phenotype in primary neuroblastoma [26]. Coco et al. reported the novel c.3605delG as the first nonsense variant found in the *ALK* gene and the only variant reported in medulloblastoma at that time [27]. Later, Trubicka et al. identified a second novel inherited *ALK* variant p.M1199L in medulloblastoma [28].

Variant c.1572del p. (Asp525MetfsTer10) that we detected in the *ALK* gene in our medulloblastoma patient #1 is a nonsense mutation located in exon 8 in the MAM domain. The MAM domain has an adhesive function, playing a role in homodimerization. It has been reported that certain variants in the MAM domain result in altered stability and activity of ALK protein [29]. This indicates that these domain–domain interactions are critical for the structure and function of the enzyme. Since we detected a nonsense mutation that creates a stop codon in our medulloblastoma patient #1, we can speculate that this variant produces a protein with impaired capability for playing its adhesive function.

In patients #2 and #3, we found missense mutations c.2543C>T (p.Ala848Val) and c.3115G>A p. (Val1039Met), respectively, considered to be variants of unknown significance.

As already mentioned, ALK protein might have a role in the development of medulloblastoma. Furthermore, Bu et al. reported a series of high-grade glioma patients with germline variants in multiple regions of the *ALK* gene, suggesting an important part in glioma formation and a potential target for therapy [30].

### 3.2. APC Gene

Adenomatous polyposis coli (APC) is a tumor-suppressor protein that induces the degradation of oncogenic beta-catenin and negatively regulates Wnt signaling [31]. It has roles in regulating cell migration, DNA replication/repair, mitosis and apoptosis [32]. Wild-type APC protein is expressed in the central nervous system and is significantly involved in the initiation of neuronal differentiation [33,34]. Also, Wnt signaling proteins regulate crucial normal brain developmental processes [35,36], including cellular adhesion and synaptic rearrangements [37]. The Wnt pathway has been involved in tumor genesis and, lately, in brain tumor genesis as well [38]. APC protein has been related to certain syndromes, such as Turcot’s syndrome, which involves the development of primary brain tumors like medulloblastomas and gliomas [39].

In our high-grade glioma patient #4, we detected p.Arg564Ter, a stop-gained pathogenic variant, which is located in a conserved armadillo (Arm) domain of the protein. This domain has a role in the Wnt signaling pathway and cytoskeletal regulation through microtubule binding [40], which is a main function of this protein, so we speculate that this p. Arg564Ter stop-gained variant in our patient produces a protein with highly altered structure and function, contributing to tumor genesis [38]. The prognosis for medulloblastoma patients with *APC* germline alteration is quite favorable with standard-of-care treatment. However, they are prone to developing various secondary malignancies, including radiation-induced high-grade gliomas. As they occur in 1–4% of patients treated with cranial radiotherapy, the influence of the germline variants can be a subject of debate [41,42,43].

### 3.3. CHEK2 Gene

*CHEK2* (checkpoint kinase 2) is a tumor-suppressor gene located at chromosome 22q12.1 encoding checkpoint kinase CHK2 involved in the DNA damage response [44]. This multifunctional kinase is involved in key cell processes like genome maintenance, cell-cycle arrest and apoptosis. The main downstream effector of activated CHK2 is the p53 protein, but it can also interact with a multitude of substrates involved in DNA damage response [45]. This kinase performs its role as a tumor suppressor by delaying cell-cycle progression enabling DNA repair, as well as by inducing apoptosis in gnomically unstable cells. Therefore, the presence of germline variants in the *CHEK2* gene disrupting the normal function of this protein could result in an increased predisposition to cancer. The *CHEK2* gene is one of the well-known CPGs, with variants associated with the occurrence of different types of pediatric tumors [46,47].

In our study, we detected one patient with c.470T>C (p.Ile157Thr) variant (patient #5). This patient had medulloblastoma and Li–Fraumeni syndrome. Germline *CHEK2* variants in general, as well as specific variant p.Ile157Thr, have been associated with Li–Fraumeni syndrome especially in *TP53*-negative patients, as was the case in our patient with supporting cancer family history [48,49]. Regarding pediatric brain tumors, the same missense variant was reported in patients with medulloblastoma, neuroblastoma and pilocytic astrocytoma [46,50]. The p.Ile157Thr *CHEK2* variant has been defined as a common variant of this CPG [51]. It has even been described as a founder variant in Slavic and German populations, occurring in 5% and 2%, respectively [52,53,54]. Based on the data so far, this is a common, low-penetrance variant of the *CHEK2* multiorgan CPG [55,56,57].

**Figure 2:**
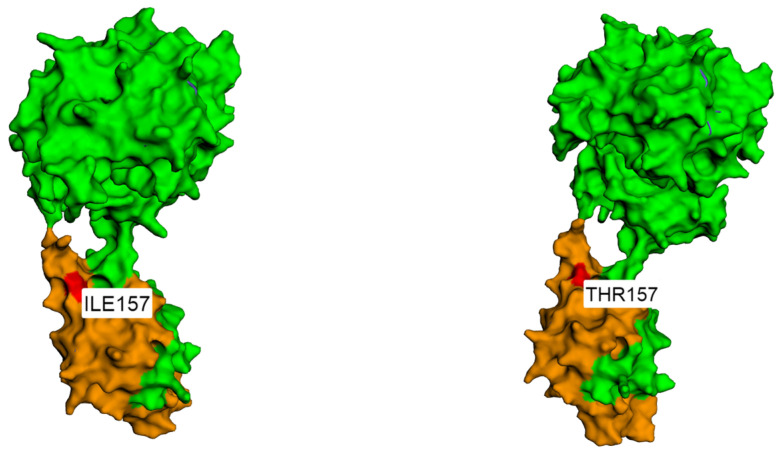
The left figure shows the protein structure of wild-type (wt) *CHEK2*, with brown representing the forkhead-associated (FHA) domain (p.112–p.191) and red representing p.Ile157. The right figure shows the protein structure of mutated (mut) *CHEK2*, with brown representing the FHA domain (p.112–p.191) and red representing changed p.Thr157 [61].

The *CHEK2* p.Ile157Thr variant is located in the forkhead-associated (FHA) domain of the CHK2 kinase region that is participating in the activation/auto-phosphorylation process [58,59]. Given that there are conflicting data about the clinical relevance that this p.Ile157Thr variant has, with the help of the bioinformatic tools, we created protein models for both wild-type and mutated CHEK2 protein, with the intention of contributing to the characterization of this variant (Figure 2). Although in silico prediction indicates a potentially damaging effect of this variant, the latest functional analyses do not support this, suggesting that the protein remains functional to the greatest extent [46,60]. Still, according to the current ACMG guidelines, *CHEK2* p.Ile157Thr is characterized as likely pathogenic.

### 3.4. ELP1 Gene

ELP1 protein is the largest subunit of the evolutionary conserved Elongator Complex, whose main function is tRNA modification and ensuring a correct translational elongation [62]. Germline loss-of-function (LOF) variants in *ELP1* have recently been strongly associated with medulloblastoma in pediatric age, predisposing a patient to tumor development in combination with constitutive activation of Sonic Hedgehog (SHH) signaling [63]. The cerebellum is described as the site of greatest ELP1 expression during brain development [64], and according to Waszak et al., one of the three consecutive mutational events probably required for the development of ELP1-associated SHH-medulloblastoma is a heterozygous germline *ELP1* LOF variant [63]. Also, in pediatric SHH-medulloblastoma, germline alterations of the *ELP1* gene have been described in 14% of cases, making this gene the most frequent genetic predisposition in medulloblastoma.

In our cohort of pediatric brain tumor patients, we detected three variants in the *ELP1* gene. In patient #6 with high-grade glioma previously treated for medulloblastoma and patient #7 with medulloblastoma, detected variants were both null variants (splice donor c.1908+1G>T and frameshift indel p.(Leu651TyrfsTer3), respectively). Splice donor c.1908+1G>T is located in the evolutionarily conserved region of the protein. The discovered variant most probably plays a role in the development of the medulloblastomas in these patients. However, the impact of the *ELP1* variant on the appearance of high-grade glioma cases is not so straightforward due to cranial irradiation, a known risk factor for secondary tumors of this type [43].

Another variant detected was in patient #8 (low-grade glioma), which was a missense p.Pro832Leu variant, with a pathogenic moderate MetaRnn in silico prediction. Current data suggest that the loss of even a single subunit of the protein causes the dysregulation of the Elongator Complex with consequent proteome instability. Interestingly, Waszak et al. found a strong association between germline LOF variants in the *ELP1* and SHH-medulloblastoma subgroup [63], so there is a recommendation that SHH-medulloblastoma patients should be analyzed for germline *ELP1* variants, in particular those presenting outside of infancy [65]. However, no association between the germline *ELP1* variant and pilocytic astrocytoma has been described in the literature so far. Therefore, this association requires further studies.

### 3.5. MLH1 and MSH2 Genes

MutL homolog 1 (*MLH1*) and mutS homolog 2 (*MSH2*) are two of the four mismatch repair (MMR) genes, together with postmeiotic segregation increased 2 (*PMS2*) and mutS homolog 6 (*MSH6*). The main role of the MMR mechanism is to correct errors that occur during the DNA replication process. The presence of germline homozygous (or compound heterozygous) mutations in MMR genes causes constitutional mismatch repair deficiency (CMMRD) syndrome [66]. CMMRD is an autosomal recessive disorder that results in the early onset of different types of tumors in early age, among them brain tumors [67]. Heterozygous MMR germline mutations are the cause of Lynch syndrome or hereditary nonpolyposis colorectal cancer (HNPCC) [68]. The development of tumors in these patients is enabled by the somatically acquired second mutation that has to be present in the tumor tissue but may not be present elsewhere. Patients with Lynch syndrome most often develop colorectal cancer, followed by endometrial cancer, ovarian cancer, breast cancer and brain tumors [69]. Genotype–phenotype analysis showed that brain neoplasms have the strongest association with *MSH1* and *MSH2* variants [70,71].

We discovered two different alterations in the *MLH1* gene in our patient #9. One (c.1611del p.(Gln537HisfsTer54)) is considered to create a premature translational stop signal and is classified as pathogenic [72]. Another one is a missense mutation (c.1613G>T p.(Trp538Leu)) considered to be a variant of unknown significance. Nevertheless, the occurrence of high-grade glioma suggests an important role of this variant in the development of the neoplasm, and these compound heterozygous mutations indicate CMMRD syndrome. The colorectal carcinoma diagnosed at a young age in the father of the patient supports the syndromic diagnosis.

*MSH2* is a tumor-suppressor gene located at chromosome 2p21. Patient #10 with the *MSH2* c.274C>G (p.Leu92Val) variant was diagnosed with ependymoma. This variant is located in the functionally relevant N-terminal domain of the protein, but it has been categorized as a variant with uncertain significance (VUS) by the ACMG guidelines. The same variant was described by Taeubner et al. in a child with CMMRD and medulloblastoma, where it was associated with another MMR variant (MSH6 p.Val809del). The authors declared *MSH2* p.Leu92Val mutation as a VUS but concluded that this variant is unlikely to be responsible for the phenotype of the patient [73].

In our study, we detected another *MSH2* variant in patient #11 with high-grade glioma. This frameshift variant (c.2382dup p.(Pro795ThrfsTer4)) is a null mutation, located in the ATP-binding domain of the protein, and has been categorized as likely pathogenic. This patient had a positive family history considering both mother’s parents consistent with Lynch syndrome [74].

### 3.6. NF1 Gene

Neurofibromatosis type 1 is a cancer predisposition syndrome showing an increased risk for the development of brain tumors [75]. This syndrome is caused by inherited or de novo germline mutations in the *NF1* gene, and it is inherited in an autosomal dominant way. The gene is located at chromosome 17q11.2 and encodes neurofibromin, a guanosine triphosphate (GTPase)-activating protein (GAP) for RAS [76]. Neurofibromin acts as a tumor-suppressor inhibiting RAS, the most prevalent proto-oncogene in all types of tumors. Loss of function of neurofibromin induces the activation of RAS signaling and its downstream pathways like mitogen-activated protein kinase/extracellular signal-regulated kinases (MAPK/ERK) and phosphatidylinositol 3-kinase/protein kinase B/mechanistic target of rapamycin (PI3K/AKT/mTOR) pathway, resulting in increased proliferation and cell growth [77].

In recent years, more than 3000 different genetic variants in the *NF1* gene have been reported, and most of them lead to loss of expression or synthesis of non-functional neurofibromin [78]. This increase in the amount of genetic data has led to numerous studies aimed at *NF1* genotype–phenotype correlation [79,80,81,82,83]. Some of these studies reported age-dependent manifestations of some cancers like optic pathway gliomas that are associated with younger pediatric NF1 patients [84]. Namely, the most prevalent type of brain tumor associated with NF1 is astrocytoma. In the pediatric population, these gliomas are most commonly localized in the optical nerve and brainstem [85].

In our study, we detected patient #12 diagnosed with a brainstem glioma to have c.3974+1G>A, a splice donor site alteration, disrupting the splicing site at the end of exon 29 of the *NF1* gene. This variant is a null mutation, pathogenic, associated with the phenotype of neurofibromatosis type 1. The same variant was reported by Tsipi et al. and, as in our case, was designated as pathogenic by the ASCG criteria [86].

### 3.7. NF2 Gene

Although it shares its name with NF1, neurofibromatosis type 2 (NF2) is a completely different clinical entity. NF2 is a cancer predisposition syndrome caused by the presence of mutations in the *NF2* gene [87]. This gene is located at chromosome 22q12.2 and encodes tumor-suppressor protein merlin, a moesin–ezrin–radixin-like protein. NF2, also called NF2-schwannomatosis, is a completely penetrant autosomal dominant condition characterized by the development of bilateral vestibular schwannomas and also ependymomas and meningiomas [88]. Only 50% of NF2 patients have a family history, i.e., they inherited the condition, while others have de novo mutations, with 60% of them being mosaics [89]. Merlin performs its role as a tumor suppressor by regulating cell proliferation in response to adhesive signaling by activating anti-mitotic signaling and, also, by inhibiting oncogenic gene expression [90]. Merlin has an inhibitory effect on multiple receptor tyrosine kinases (RTK)-like receptors belonging to the ErbB/EGFR receptor family, platelet-derived growth factor receptor (PDGFR), insulin-like growth factor 1 receptor (IGF1R), and vascular endothelial growth factor receptor (VEGFR) [91]. Mutations affecting the *NF2* gene that causes inactivation of merlin lead to activation of RTK downstream pathways like PI3K/AKT/mTORC1 and RAS [92,93,94,95]. These findings indicate that both neurofibromin in NF1 and merlin in NF2 share the same signaling pathways and therefore have the same therapeutic targets [96].

Numerous different *NF2* variants have been identified so far, and certain regularities between the genotype and the clinical manifestations of NF2 were established [96,97,98]. This knowledge has led to the definition of the UK NF2 Genetic Severity Score, which has been reevaluated and improved over the years [99,100,101]. In our study, we detected one patient (#13) with NF2 and germinative variants in *NF2* c.999 +1G>A (end of exon 10). It is a splice variant by type, null mutation, defined as pathogenic by ASCG criteria and causing moderate-to-severe clinical manifestations, since splice mutations occurring between exon 8 and 13 are qualified as moderate to severe by NF2 Genetic Severity Score criteria. This correlates with the severity of the clinical presentation of our patient (bilateral vestibular schwannomas, multiple ependymomas and meningiomas with distinct neurological sequelae, the most prominent being moderate hearing loss and paraplegia).

### 3.8. TP53 Gene

The tumor-suppressor gene *TP53*, known as the guardian of the genome, encodes the p53 protein, which has an important role in the cell cycle by keeping cell division under control. When DNA damage occurs, p53 is activated, it binds to DNA as a tetrameric transcription factor and regulates gene expression, which blocks further progression through the cell cycle [102]. Also, p53 plays a role in senescence, apoptosis, differentiation, autophagy, metabolism and angiogenesis. These multiple roles p53 are achieved through direct regulation of hundreds of different genes [103]. Clinical and experimental analysis indicates that the loss of p53 function is a key initial event in glioma development, together with other genetic and epigenetic alterations [104]. *TP53* is one of those markers that are diagnostically or prognostically proven to be important in glioma tumorigenesis [105]. Mutations in *TP53* occur early in glioma progression and are mostly missense mutations that lead to overexpression of the p53 protein in the cells [106]. P53 can block cell-cycle progression and induce morphological changes resembling differentiation in glioma cell lines [107]. Sarma et al. described a prevalent pattern of *TP53* point mutations in glioma patients and showed their relevance in glioma genesis. They state that when located in the DNA-binding domain, these mutations can alter p53 protein conformation and function, which can lead to altered downstream signaling [108].

**Figure 3:**
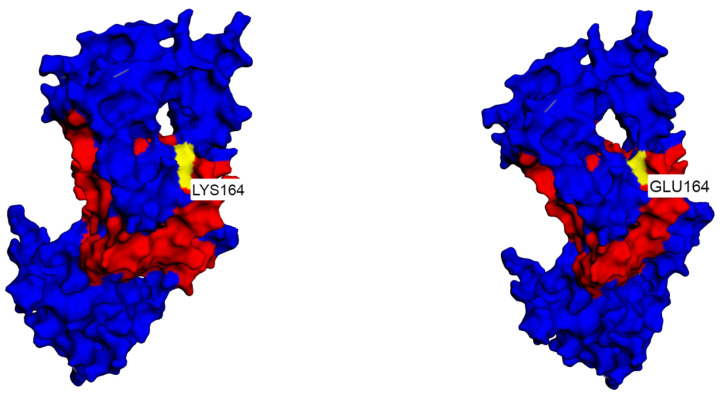
The left figure shows the protein structure of wild-type (wt) p53, with red representing the DNA-binding domain (p.100–p.288) and yellow representing p.Lys164. The right figure shows the protein structure of mutated (mut) p53, with red representing the DNA-binding domain (p.100–p.288) and yellow representing changed p.Glu164 [61].

In our high-grade glioma patient #14, we detected the p.(Lys164Glu) missense likely pathogenic variant, which is located in the DNA-binding domain of the protein. Although this p.(Lys164Glu) variant is reported in the literature and classified as LP, there is still not enough clear evidence of its clinical impact. For that reason, with the help of the bioinformatic tools, we created protein models for both wild-type and mutated p53 protein, with the intention of contributing to the characterization of this variant (Figure 3). It has a MetaRnn in silico prediction of strong pathogenic. Given the above-reported data from the literature, we can assume that this p.(Lys164Glu) variant could represent one of the key initial events in glioma development by disrupting the protein’s DNA-binding ability and resulting in the loss of its tumor suppressive capability. High-grade gliomas that occur in patients with germline *TP53* mutation have a variable prognosis. However, adaptation of an oncologic approach, especially radiotherapy, is advised, along with close surveillance for other malignancies [9,109,110].

### 3.9. Detecting Germline Variants in the Emerging Era of Precision Medicine

Precision medicine is based on finding specific genomic variants significant for tailoring a personalized plan for a single patient. This approach is thoroughly explored in pediatric oncology, and treatment dogma recently started shifting from treating specific tumor types to targeting actionable genomic alterations [111,112]. An excellent example in pediatric neuro-oncology is the use of trametinib (MEK inhibitor) and dabrafenib (BRAF inhibitor) in patients with *BRAF* V600-mutant low-grade glioma, which exhibited a better overall response rate compared to standard chemotherapy regimen [113]. Further findings resulted in the approval of these drugs in 2023 by the Food and Drug Administration (FDA) for use in pediatric patients with this diagnosis. Even though patients with NF1 low-grade gliomas share the same tumor pathways with *BRAF* V600-mutant low-grade gliomas, the administration of these drugs is still mostly limited to clinical trials or off-label use. Selumetinib, another MEK inhibitor, has been registered for children with symptomatic inoperable plexiform neurofibromas. Since patients with NF1 share the same molecular pathway changes, the empiric use of these drugs is currently being evaluated by various clinical trials. Even though the standard of care for low-grade gliomas in NF1 patients is chemotherapy (carboplatin, vincristine), these targeted therapies might become the first-line approach in the following years [114,115]. For our patient number #12 with NF1, the use of targeted therapy is reserved for potential disease progression.

Knowledge about the same signaling pathways in NF2 implies similar targeted therapy. Nevertheless, mostly VEGF and mTOR inhibitors are investigated as effective targeted therapies for this syndrome [96,116]. We achieved disease control with everolimus (mTOR inhibitor) and bevacizumab (anti-VGEF antibody) in our patient #13 with NF2, showing the importance of targeted therapies in genetic disorders. Therefore, *NF1* and *NF2* genes are considered actionable in terms of selecting appropriate treatment for the patients.

Aside from *NF1* and *NF2* genes, *CHEK2* and *TP53* variants, heralding Li–Fraumeni syndrome, are actionable in terms of tailoring specific surveillance plans for these patients who harbor a high risk for cancer development throughout life. Additionally, avoidance or dose modification of radiotherapy is important in these patients [9,48,117]. Nevertheless, both patients with these germline mutations (#5 with medulloblastoma and #14 with high-grade glioma) displayed extremely aggressive tumors with dismal prognoses exhibiting progression to all treatment modalities including high-dose chemotherapy in patient #5. Radiotherapy was administered routinely. Routine germline testing of pediatric CNS patients in our country would result in knowledge of these syndromes and timely guide decision-making. In a comprehensive study by Akhavanfard S et al. on germline genomic variants in children with solid tumors, *CHEK2* was recognized as a known CPG with pathogenic/likely pathogenic variants that can be targeted with FDA-approved drugs [118]. Clinical trials of sunitinib (NCT01462695) and gefitinib (NCT00042991) in glioma patients showed discouraging results. Similar studies in patients with medulloblastoma have not been executed, and preclinical data imply *CHEK2* alterations as a potential target in this disease [119].

The use of ALK inhibitors in children has been discussed by the Second Pediatric Strategy Forum for anaplastic lymphoma kinase (ALK) inhibition in pediatric malignancies, especially emphasizing ensartinib, a second-generation ALK inhibitor with good CNS penetrance [120]. The Pediatric MATCH Screening Trial (NCT03155620) is currently recruiting children with recurrent solid malignancies, including high-grade gliomas with *ALK* alterations, to evaluate the effects of this novel drug. Interestingly, patient #2 developed postoperative mutism, a complication without evident predictive factors. Currently, there is a clinical trial (NCT02300766) aiming to define these factors, and one of the plausible explanations is genomic differences between patients.

Furthermore, *MLH1* and *MSH2* variants heralding Lynch syndrome or CMMRD syndrome are also considered actionable in a way of further surveillance and potential use of immune checkpoint inhibitors. High-grade gliomas with MMR deficiencies are recognized to confer poorer prognosis due to resistance to the current standard-of-care chemotherapy (temozolomide) but are considered to respond better to novel therapies like immune checkpoint inhibitors [121,122]. There is currently an open phase I clinical trial of pembrolizumab in younger patients with brain tumors, especially high-grade gliomas (NCT02359565). Furthermore, preclinical studies show promising results of histone deacetylase inhibition with quisinostat [123].

## 4. Materials and Methods

### 4.1. Patients

The study cohort consisted of 51 consecutive pediatric CNS tumor patients (aged 0–18 years) diagnosed and treated in the National Cancer Research Center, Belgrade, Serbia (*Institut za onkologiju i radiologiju Srbije*) between July 2021 and June 2023. All pediatric neuro-oncology patients requiring therapy after surgery in Serbia are treated in this tertiary center. Our cohort also encompassed a majority of affected children from Republika Srpska and Brčko District (Bosnia and Herzegovina) who were referred to our institution. The study was conducted according to the institutional ethical policies (Ref. No. 01-1/2023/2081). Written informed consent was obtained from parents/guardians of all participants before the beginning of medical treatment.

Clinical data (sex, age at diagnosis, date of symptom onset, date of diagnosis, tumor pathology, tumor location, family history and physical examination findings) were collected through history taking and physical examination. The total diagnostic interval (TDI) was calculated as the difference between the date of diagnosis (date of surgery for children who underwent it or date of first MRI indicating tumor presence in inoperable patients) and the date of symptom onset presented in weeks [124]. The necessity for further genetic analysis was estimated with the Jongmans et al. selection tool [12], which takes into consideration family history, tumor pathology, presence of multiple tumors (synchronous or metachronous), congenital anomalies or specific symptoms and excessive treatment toxicity. The necessity for further genetic analysis was also evaluated by the McGill Interactive Pediatric OncoGenetic Guidelines (MIPOGG), a follow-up pathway that considers most of the aforementioned referral factors [125].

### 4.2. Genetic Testing and Data Analysis

Peripheral blood samples were harvested from all patients, and Genomic DNA was isolated using a QIAamp DNA Blood-Mini-Kit (Qiagen, Hilden, Germany), according to the manufacturer’s instructions. To detect the presence of germline variants in CPGs, we analyzed 51 patients using the NGS approach and Clinical Exome Sequencing TruSight One Gene Panel (Illumina, San Diego, CA USA). This panel includes all the known disease-associated genes described in the OMIM database until 2013, designed to cover all exons and flanking intronic regions of 4813 genes (approx. 62,000 exons). Bioinformatic analysis was done using an “in-house” gene list panel which included 144 genes related to pediatric brain tumors (Table S1: “In-house” gene list panel), complemented with the gene list panel Neoplasm (HP:0002664) consisting of 837 genes, in which are detected pathogenic, likely pathogenic and variants of uncertain significance (VUS). The “in house” gene list panel was designed according to the already described germline gene variants that confer greater risk for CNS tumor development and gene variants that are described as predisposing to other cancers, with their somatic alterations occurring in CNS tumor tissue [3,5,18,21,30,126,127,128,129,130,131,132,133,134,135,136].

Systemic interpretation of variants was performed using Variant Interpreter (Illumina). Variants were classified according to the recommendations of the American College of Medical Genetics and Genomics (ACMG) [137], ClinVar database [138] and Cosmic database [139].

### 4.3. Statistical Analysis

Descriptive statistics and analysis of the total diagnostic interval according to the presence of variants using the double-sided Mann–Whitney U test were performed using the software package EZR v.1.54 (Saitama Medical Centre, Jichi Medical University, Saitama, Japan) [140]. A *p*-value of <0.05 was considered statistically significant. Numeric results were presented as median with interquartile range.

## 5. Conclusions

This study represents the first comprehensive evaluation of germline variants in pediatric patients with CNS tumors in the Western Balkans region. We described variants in several CPGs, namely *ALK*, *APC*, *CHEK2*, *ELP1*, *MLH1*, *MSH2*, *NF1*, *NF2* and *TP53*. Not only do our results contribute to the understanding of the genetic basis of pediatric CNS tumors, they also emphasize the importance of the timely discovery of alterations in CPGs in clinical practice and decision-making. Detection of germline variants is also very important in genetic counseling in identifying family members at risk of developing neoplasm and developing future surveillance plans for all of them. Lastly, information about the presence of germline variants in CPGs could influence the decision on the therapeutic protocol, making it, in the true sense, personalized for each individual patient.

## Figures and Tables

**Table 1:** Patient characteristics.

Characteristics	Absolute Numbers	%
**Sex**		
Male	29	57%
Female	22	43%
**Tumor pathology**		
High-grade glioma (HGG)	15	29%
Medulloblastoma	11	21%
Low-grade glioma (LGG)	8	16%
Ependymoma	3	6%
Atypical teratoid rhabdoid tumor (ATRT)	2	4%
Choroid plexus tumor (CPT)	2	4%
Craniopharyngioma	1	2%
Germ cell tumor (GCT)	1	2%
Diffuse glioneuronal leptomeningeal tumor	1	2%
Other ^1^	2	4%
Unknown ^2^	5	10%
**Secondary malignancies**	3	6%
**Lethal outcome**	7	14%
**Jongmans et al. tool suggestions for genetics exploration**	29	57%
**MIPOGG suggestions for genetics exploration**	39	76%
**Patients with germline variants in predisposing genes**	14	27%

^1^ Other tumor types included one patient with composite tumor (ependymoma, low-grade glioma) and one patient with multiple tumors (ependymomas, vestibular schwannomas, meningiomas); ^2^ Unknown tumor types encompassed one optic pathway glioma, three diffuse intrinsic pontine gliomas and one secondary malignancy suggestive of high-grade glioma.

**Table 2:** Variants in cancer predisposition genes.

Pt. No.	Sex	Age	Location	Type	Gene	Variant	Zygosity	Clin Var	Family History	Radiation Therapy	Systemic Therapy	Other Relevant Data
1	m	11	IT	MBL	*ALK*	NM_004304.4 c.1572del p.(Asp525MetfsTer10)	HZ	FSI LP	BT (12.5%)	CSI 35.2 Gy/22 + FCP boost 19.8 Gy/11	VCR, Cis, CCNU	
2	m	4	IT	MBL	*ALK*	NM_004304.4 c.2543C>T (p.Ala848Val)	HZ	MS VUS	*LC (12.5%), LrC (12.5%)*	?	VCR, Cyc + ?	postoperative mutism
3	m	15	ST	HGG	*ALK*	NM_004304.4 c.3115G>A p.(Val1039Met)	HZ	MS VUS	/	59.4 Gy/33	TMZ	
4	f	13	BS	HGG	*APC*	NM_000038.5 c.1690C>T p.(Arg564Ter) rs137854574	HZ	SG (NV) P	FAP (50%), *BC (25%)*	(MBL) CSI 23.4 Gy/13 + FCP boost 30.6 Gy/17	VCR, Cis, CCNU, TMZ	treated for MBL
5	m	2	IT	MBL	*CHEK2*	NM_007194.3 c.470T>C p.(Ile157Thr) rs17879961	HZ	MS LP	*LY (25%), AC (12.5%),* PC (25%)	CSI 24 Gy/15 + FCP boost 30.6 Gy/17	VCR, Cis, VP, Cyc, MTX, HDCT	
6	f	12	ST	HGG	*ELP1*	NM_003640.4 c.1908+1G>T	HZ	MS VUS	*LEU (25%)*	30 Gy/15 + 16 Gy/9	VCR, Cis, CCNU, TMZ	treated for MBL
7	f	14	IT	MBL	*ELP1*	NM_003640.4 c.1952del p.(Leu651TyrfsTer3)	HZ	FSI (NV) LP	*CRC (25%)*	CSI 23.4 Gy/13 + FCP boost 30.6 Gy/17	VCR, Cis, CCNU	hair depigmentation, VCR neuropathy
8	m	12	BS	PA	*ELP1*	NM_003640.4 c.2495C>T p.Pro832Leu	HZ	SD (NV) LP	BT (25%), 6 UT (6.25–25%)	50.4 Gy/30 + boost 3.6 Gy/2	TR, DF	
9	m	15	ST	HGG	*MLH1/* *MLH1*	NM_000249.3 c.1611del p.(Gln537HisfsTer54)/NM_000249.3 c.1613G>T p.(Trp538Leu)	HZ/ HZ	FSI. P/ MS. VUS	CRC (50%)	59.4 Gy/33	TMZ	skin hem-angioma
10	m	2	IT	EP	*MSH2*	NM_000251.2 c.274C>G (p.Leu92Val)	HZ	MS VUS	/	54 Gy/30 + 5.4 Gy/3	/	
11	f	12	ST	HGG	*MSH2*	NM_000251.2 c.2382dup p.(Pro795ThrfsTer4)	HZ	FSI (NV) LP	*SC (25%), HT (25%)*	CSI 36 Gy/20 + boost 19.8 Gy/11	TMZ	
12	m	5	BS SC	UNK	*NF1*	NM_001042492.2 c.3974+1G>A	HZ	SD (NV) P	*Fs, OPGs (25–50%)*	/	VCR, Carbo, VBL	multiple café au lait spots
13	f	11	BSSC	many	*NF2*	NM_000268.3 c.999+1G>A	HZ	SD (NV) P	PC (25%)	59.4 Gy/33	BV, EV	multiple café au lait spots
14	f	4	ST	HGG	*TP53*	NM_000546.5 c.490A>G p.(Lys164Glu) rs879254249	HZ	MS LP	*CP (50%), BrC (25%)*	54 Gy/30	TMZ	

**Sex**: m-male, f-female; **Location**: IT—infratentorial, ST—supratentorial, BS—brainstem, SC—spinal cord; **Type**: MBL—medulloblastoma, HGG—high-grade glioma, PA—Pilocytic astrocytoma with BRAF V600E mutation, AT/RT—atypical teratoid/rhabdoid tumor, EP—ependymoma, UNK—unknown, i.e., optic pathway glioma, many—multiple ependymomas and meningiomas and bilateral schwannomas; **Zygosity**: HZ—heterozygous; **ClinVar**: FSI—frameshift indels, LP– likely pathogenic, MS—missense, VUS—variant of unknown significance, SG—stop-gained, NV—null variant, P—pathogenic, SD—splice donor, NC—non coding, FS—frameshift; **Family history**: BT—brain tumor, LC—lung cancer, LrC—laryngeal cancer, FAP—familial adenomatous polyposis, BC—biliary cancer, LY—lymphoma, AC—abdominal cancer not specified, PrC—prostatic cancer, UT—tumors with unknown pathology, LEU—leukemia, LC—lung cancer, CRC—colorectal carcinoma, SC—stomach cancer, HT—malignant tumor in head region not specified, Fs—fibromas, OPGs—optic pathway gliomas, PC—pancreatic cancer, CP—colon polyp, BrC—breast cancer, *italic*—maternal lineage, coefficient of relatedness in brackets; **Radiation therapy**: CSI—craniospinal irradiation, otherwise local radiation with boost, FCP—posterior fossa, p—proton beam radiotherapy, otherwise X-ray radiotherapy, ?—continued treatment abroad; **Systemic therapy**: VCR—vincristine, Cis—cisplatin, CCNU—lomustine, TMZ—temozolomide, VP—etoposide, Cyc—cyclophosphamide, MTX—methotrexate, Carbo—carboplatin, HDCT—high-dose chemotherapy with carboplatin and thiotepa, TR—trametinib, DF—dabrafenib, BV—bevacizumab, EV—everolimus, VBL—vinblastine, ?—continued treatment abroad.

**Table 3:** Comparison of recent studies of germline variants in pediatric neuro-oncology cohorts. The upper part of the table shows genes with germline variants found in particular tumor types reported by one of the studies presented in the lower part of the table.

Genes with Reported Germline Variants in Particular Tumor Types											
Gene	ATRT	MBL	PBL	LGG	HGG	EP	GCT	MN	VS	UKN	NR
*ALK*											
*APC*					*						
*CHEK2*											
*DICER1*											
*ELP1*											
*FANCI*											
*KDM4C*											
*MLH1*											
*MSH2*											
*MSH6*											
*NF1*											
*NF2*											
*PTCH1*											
*SMARCB1*											

*TP53*											
*TSC1*											
*TSC2*											
*VHL*											
*WRN*											
**Recent NGS studies of germline variants in pediatric neuro-oncology cohorts**											
**Study**		**Color**	**Patient number**	**Sampling remarks**							
Barsan et al. (2019) [19]			58	non-consecutive patients who underwent NGS on clinical oncologist’s demand							
Fukushima et al. (2022) [21]			38	consecutive patients from a single center, mostly germ cell tumors							
Diaz de Ståhl et al. (2023) [20]			82	purposive sampling from the national biobank to represent major tumor type frequencies							
Jovanović et al. (2023) [this article]			51	consecutive patients from the national tertiary referral center							

ATRT—atypical teratoid/rhabdoid tumor, MBL—medulloblastoma, PBL—pineoblastoma, LGG—low-grade glioma, HGG—high-grade glioma (* secondary tumor), EP—ependymoma, GCT—germ cell tumor, MN—meningioma, VS—vestibular schwannoma, UKN—unknown, NR—not reported.

## Data Availability

The data presented in this study are available within the article and Supplementary Materials; further inquiries can be directed to the corresponding author.

## Supplementary Materials

The following supporting information can be downloaded at: https://www.mdpi.com/article/10.3390/ijms242417387/s1.
