# Supplementary material for: Germline Variants in Cancer Predisposition Genes in Pediatric Patients with Central Nervous System Tumors
Source: Int J Mol Sci. 2023 Dec 12;24(24):17387. doi: 10.3390/ijms242417387 (PMC10744041; doi:10.3390/ijms242417387)
Supplement: Supplementary file 1 [file ijms-24-17387-s001.zip › In house PBT gene list.pdf]

“In-house” Pediatric Brain Tumor gene list (genes that are on TSO platform)

ACD
ALK
APC
ATM
ATRX
BAP1
BLM
BMPR1A
BRAF
BRCA1
BRCA2
BRIP1
BUB1B
CASP9
CBL
CCND1
CDC73
CDH1
CDK4
CDKN1B
CDKN2A
CDKN2BAS1
CEBPA
CHEK2
CREBBP
CTC1
DCLRE1B
DDB2
DHCR7
DICER1
DIS3L2
DKC1
EGFR
ELP1
EP300
EPCAM
ERCC2
ERCC3
ERCC4
ERCC5
ETFA
FANCA
FANCB
FANCC
FANCD2
FANCE
FANCF
FANCG
FANCI
FANCL
FANCM
FH
GATA2
GFRA1
GJB2
GLI3
GSTO1
GSTP1
GSTT1
HNF4A
HRAS
IDH1
IDH2
IGFBP3
IL4
IL4R
KRAS
LIG1
LIG4
MAP2K1
MAP2K2
MAX
MDM2
MDM4
MED13L
MEN1
MGMT
MLH1
MSH2
MSH3
MSH6
MTHFR
MUTYH
NBN
NEFL
NF1
NF2
NOP10
NOTCH2
NRAS
NSD1
PALB2
PAX5
PIK3CA
PMS2
POLH
PPM1D
PRKAR1A
PRKDC
PTCH1
PTCH2
PTEN
PTPN11
RAD51
RAD51C
RAF1
RB1
RECQL4
RET
RRM2B
RUNX1
SAMD9
SBDS
SDHA
SDHAF2
SDHB
SDHC
SDHD
SHOC2
SLX4
SMAD4
SMARCA4
SMARCB1
SMARCE1
SOS1
STEAP3
STK11
SUFU
TERT
TINF2
TMEM127
TP53
TPP1
TSC1
TSC2
UBE2T
VHL
WRN
WT1
XPA
XPC
XRCC1
XRCC2
XRCC3
